# Target users’ acceptance of a pharmacist-led prescribing service for pre-exposure prophylaxis (PrEP) for human immunodeficiency virus (HIV)

**DOI:** 10.1177/17151635231177027

**Published:** 2023-06-12

**Authors:** Calum B. MacDonald, Andrea L. Murphy, Jennifer E. Isenor, Tasha D. Ramsey, Kirk Furlotte, Alesha J. Smith, Andrea Bishop, Deborah V. Kelly, Lisa Woodill, Connor Booker, Kyle John Wilby

**Affiliations:** Faculty of Medicine, Dalhousie University, Halifax, Nova Scotia; College of Pharmacy, Faculty of Health, Dalhousie University, Halifax, Nova Scotia; College of Pharmacy, Faculty of Health, Dalhousie University, Halifax, Nova Scotia; College of Pharmacy, Faculty of Health, Dalhousie University, Halifax, Nova Scotia; Pharmacy Department, Nova Scotia Health Authority, Halifax, Nova Scotia; Community-Based Research Centre – Atlantic Region, Halifax, Nova Scotia; School of Pharmacy, University of Otago, Dunedin, New Zealand; College of Pharmacy, Faculty of Health, Dalhousie University, Halifax, Nova Scotia; Nova Scotia College of Pharmacists, Halifax, Nova Scotia; School of Pharmacy, Memorial University of Newfoundland, St. John’s, Newfoundland and Labrador; Pharmacy Association of Nova Scotia, Halifax, Nova Scotia; College of Pharmacy, Faculty of Health, Dalhousie University, Halifax, Nova Scotia; College of Pharmacy, Faculty of Health, Dalhousie University, Halifax, Nova Scotia

## Abstract

**Background:**

Pre-exposure prophylaxis (PrEP) for human immunodeficiency virus (HIV) is a highly effective way to reduce virus transmission. There have been increasing calls to improve access to PrEP in Canada. One way to improve access is by having more prescribers available. The objective of this study was to determine target users’ acceptance of a PrEP-prescribing service by pharmacists in Nova Scotia.

**Methods:**

A triangulation, mixed-methods study was conducted consisting of an online survey and qualitative interviews underpinned by the Theoretical Framework of Acceptability (TFA) constructs (affective attitude, burden, ethicality, intervention coherence, opportunity cost, perceived effectiveness and self-efficacy). Participants were those eligible for PrEP in Nova Scotia (men who have sex with men or transgender women, persons who inject drugs and HIV-negative individuals in serodiscordant relationships). Descriptive statistics and ordinal logistic regression were used to analyze survey data. Interview data were deductively coded according to each TFA construct and then inductively coded to determine themes within each construct.

**Results:**

A total of 148 responses were captured by the survey, and 15 participants were interviewed. Participants supported pharmacists’ prescribing PrEP across all TFA constructs from both survey and interview data. Identified concerns related to pharmacists’ abilities to order and view lab results, pharmacists’ knowledge and skills for sexual health and the potential for experiencing stigma within pharmacy settings.

**Conclusion:**

A pharmacist-led PrEP-prescribing service is acceptable to eligible populations in Nova Scotia. The feasibility of PrEP prescribing by pharmacists should be pursued as an intervention to increase access to PrEP.

**Knowledge into Practice**

- This study found that pharmacist prescribing of pre-exposure prophylaxis (PrEP) for human immunodeficiency virus (HIV) in Nova Scotia is acceptable for eligible populations.
- Target users believe having pharmacists prescribe PrEP will increase access to this medication.
- The findings have implications for pharmacists’ scope of practice in Canada.

## Introduction

Pre-exposure prophylaxis (PrEP) for human immunodeficiency virus (HIV) is a highly effective way to minimize HIV transmission within high-risk populations.^1-5^ Preventing HIV is an important public health goal. In 2020, the new diagnosis rate of HIV in Canada was 4.3 per 100,000, and 1 in 10 individuals living with HIV in Canada remains undiagnosed.^6,7^ PrEP therapy consists of providing patients a combination of antiretrovirals in combination with education and counselling for safer sex practices.^8^ Patients taking PrEP are also encouraged to test for HIV and other sexually transmitted infections every 3 months for ongoing monitoring.^8^ PrEP therapy therefore requires consistent access to care. Underserved populations (men who have sex with men [MSM] or transgender women [TGW], persons that inject drugs) may also experience additional benefits from receiving PrEP therapy through a connection to a point of access to health care.^8^ Several of these groups also have higher rates of HIV, with estimates of 239.4 per 100,000 for people who inject drugs and 166.2 per 100,000 for sexually active gay or bisexual individuals and MSM.^7^

There have been recent calls to increase PrEP access for Canadians.^1^ Although the prevalence of PrEP users increased 4-fold in Canada between 2016 and 2020, access issues remain.^9^ One of the major barriers to access is the availability of capable prescribers who are knowledgeable about the medications, laboratory requirements and education for safer sex practices. In Canada, 70% of PrEP prescriptions are written in the primary care setting, thus requiring these providers to be capable of PrEP prescribing and monitoring.^9^ Access may also be limited from patients’ health care avoidance behaviours, known to influence how those from underserved communities access care.^10^ For example, a patient with past experiences of discrimination or stigma within health care settings may choose to delay or avoid future interactions, which may contribute to population-level health disparities.^11^ Providing patients with increased and convenient access to PrEP prescribers within their own communities may help to improve PrEP uptake, link these patients with the health care system and reduce avoidance behaviours.^8,10^ In addition, increasing the visibility of PrEP access through more providers in primary care in communities can facilitate decreasing stigma around its use.^12^

**Mise En Pratique Des Connaissances**

- Cette étude a montré que la prescription par les pharmaciens de la prophylaxie pré-exposition (PrEP) pour le virus de l’immunodéficience humaine (VIH) en Nouvelle-Écosse est acceptable pour les populations admissibles.
- Les utilisateurs cibles estiment que la prescription de la PrEP par les pharmaciens permettra d’améliorer l’accès à ce médicament.
- Les résultats ont des répercussions sur le champ d’activité des pharmaciens au Canada.

Pharmacists are embedded, trusted health care professionals within the communities and have a scope of practice that enables for provision of many health services in addition to and in support of medication management.^13^ In Nova Scotia, many of these services focus on prescribing, such as prescribing for conditions approved by Council (e.g., common ailments, preventative conditions, prescribing with diagnoses or under a protocol, etc.); prescribing renewals, adaptations, therapeutic substitutions, for emergencies; and prescribing Schedule II, III and unscheduled drugs.^14^ Pharmacists in Nova Scotia are also supported with a legislative framework surrounding laboratory testing.^15-17^ A PrEP-prescribing service offered by pharmacists, which includes assessment, laboratory test ordering and interpretation, prescribing and education, would increase prescriber availability for patients and would further provide patients with a consistent point of contact within the health care system. Data from other settings have shown that patients are in favour of pharmacist-managed PrEP services.^18^

Although a PrEP-prescribing service by pharmacists may have many benefits, target users’ acceptance in Nova Scotia is unknown. The Theoretical Framework of Acceptability (TFA) for health care interventions consists of 7 constructs (affective attitude, burden, ethicality, intervention coherence, opportunity costs, perceived effectiveness, self-efficacy) and can be used to examine cognitive and emotional responses to interventions.^19^ The objective of this study was to determine target users’ acceptance of a PrEP-prescribing service by pharmacists in Nova Scotia.

## Methods

### Study design

This was a triangulation mixed-methods study consisting of an anonymous, self-administered cross-sectional online survey, followed by semistructured qualitative interviews. This study was approved by the Dalhousie University Health Sciences Research Ethics Board (#2022-6044).

### Participants

Any person aged 18 years or older qualifying for Pharmacare coverage to receive PrEP in Nova Scotia was eligible to participate. Those who qualify for coverage are part of the following groups: MSM or TGW, HIV-negative heterosexual individuals in sexual relationships with someone positive for HIV or people who inject drugs. As population sizes are unknown, no formal sample size calculation was completed. Snowball sampling was used for recruitment by sharing social media posts and e-mails with community organizations (e.g., Community-based Research Centre) and advocacy groups that represented and engaged with the eligible populations, as well as social media post shares by community pharmacies in Nova Scotia.

### Questionnaire and interview guide development

The questionnaire was underpinned by the TFA.^19^ A literature review identified studies of patient perceptions of pharmacist-led PrEP prescribing. Questionnaire items were developed from the literature and organized under each TFA construct and evaluated for relevance. Additional items were created and categorized by investigators for each TFA construct. The final questionnaire was piloted for face and content validity with the research team and 2 potential participants who made minor wording changes. The final survey had 21 items, including 6 demographic and 15 Likert-type (*strongly disagree, disagree, neutral, agree and strongly agree* and *prefer not to answer*) items related to the TFA (affective attitude *n* = 2, burden *n* = 3, ethicality *n* = 2, intervention coherence *n* = 1, opportunity cost *n* = 3, perceived effectiveness *n* = 2, self-efficacy *n* = 2).

The interview guide included 7 main questions for each TFA construct. The guide was piloted with 2 interviewees, and responses were included in the analysis. Probing occurred when the interviewer deemed that elaboration of the responses was required.

### Data collection

The online survey was deployed using Research Electronic Data Capture (REDCap) from June 13 to July 19, 2022.^20,21^ As part of the survey, participants were given the option to self-identify interest in participating in an interview. Interested participants were contacted using convenience sampling (i.e., first-come basis) by 1 of the investigators (C.B.M.). Informed consent was obtained with the consent information sent via e-mail. All interviews were conducted via Microsoft Teams and were audio-recorded, then transcribed verbatim. The interviewer (C.B.M.) was trained by the primary investigator (K.J.W.) using 2 interviews before the remaining interviews were completed independently. Interviews took place until it was determined that little new information was being brought forward by participants. All interviewed participants received a $30 gift card for their time. No incentive was provided for survey respondents.

### Data analysis

Quantitative and qualitative data were analyzed separately and integrated in the determination and interpretation of results. Survey data were analyzed descriptively, and all completed and partially completed responses (respondents were allowed to skip demographic questions) were included. Responses for *strongly agree* and *agree*, as well as *strongly disagree* and *disagree*, were combined for reporting purposes into *agree* and *disagree*. Ordinal logistic regression was used to determine associations between Likert-item responses and demographic variables. All statistical analyses were conducted with SPSS v.28.

Interview recordings were deductively coded according to the 7 constructs of the TFA.^19^ Transcription and coding occurred within 5 days postinterview. Coding was completed by 1 investigator (C.B.M.) and reviewed with another (K.J.W.). Coding was conducted and iteratively reviewed with each new interview to assess the need for additional interviews. Following deductive coding using the TFA, the data were independently reviewed by 2 investigators (C.B.M., K.J.W.) and inductively coded to identify themes within each construct.^22^ Transcripts were then re-reviewed to search for supporting and disproving evidence in the form of quotes to demonstrate the validity of the data. Final themes and supporting data were agreed upon by all team members.

## Results

A total of 148 participants completed the survey. Demographic information is presented in Table 1. Most participants identified as MSM or TGW (95.8%) and lived within the Halifax Regional Municipality (82.6%). Ages ranged from 18 to 67 years, with a mean age of 32 years. Approximately 40% of the sample were current or previous PrEP users, and 59% of respondents had never used PrEP. Almost half (47.9%) of respondents were interested in PrEP to prevent HIV transmission. Many respondents (68.1%) used a pharmacist-prescribing service (any indication) prior to completing this survey.

**Table 1:** Demographic characteristics of survey respondents (*N* = 148)

Demographic*	*n* (%)
Age, years (*n* = 142)	
Mean (SD)	31.56 (7.59)
Nova Scotia PrEP eligibility criteria (participants could select more than 1 category)	
Men who have sex with men or transgender women	138 (95.8)
Heterosexual HIV-negative person in a relationship with an HIV-positive partner	3 (2.1)
Person who injects drugs	5 (3.5)
Prefer not to say	4 (2.8)
PrEP status (*n* = 144)	
Current PrEP user	39 (27.1)
Previous PrEP user	19 (13.2)
Never a PrEP user	85 (59)
Not interested in using PrEP	1 (0.7)
Interest in PrEP for HIV prevention (*n* = 144)	
Current PrEP user	38 (26.4)
Very interested	69 (47.9)
Somewhat interested	30 (20.8)
Not at all interested	5 (3.5)
Prefer not to answer	2 (1.4)
Location (*n* = 144)	
Located within Halifax Regional Municipality (HRM)	119 (82.6)
Not located within HRM	24 (16.7)
Prefer not to answer	1 (0.7)
Pharmacist prescribing experience (*n* = 144)	
Had a pharmacist prescribe medication before	98 (68.1)
Never had a pharmacist prescribe medication before	43 (29.9)
Prefer not to answer	3 (2)

HIV, human immunodeficiency virus; PrEP, pre-exposure prophylaxis.

* Respondents were allowed to skip demographic questions, if desired.

One hundred ten survey respondents self-identified as being interested in providing an interview, and 15 were selected. Each interview was approximately 15 to 20 minutes in length. Interviews were stopped after the 15 were completed, as little new information was being discussed by the participants.

Survey results according to the TFA constructs are presented in Table 2 and qualitative findings from participant interviews in Table 3. Ordinal logistic regression analyses did not result in any statistically significant findings (data not shown), suggesting participant demographics were not associated with TFA construct responses. A description of findings from both the survey and interviews, including identification of themes within each construct, is presented below.

**Table 2:** Participant perceptions of prescribing PrEP obtained from the survey and mapped to the Theoretical Framework of Acceptability constructs*

Question	TFA construct	Disagree, *n* (%)	Neutral, *n* (%)	Agree, *n* (%)
I would feel comfortable having a pharmacist prescribe and start me on PrEP.	Affective attitude	7 (4.7)	4 (2.7)	137 (92.6)
I would feel comfortable going to see a pharmacist to discuss PrEP prior to starting the medication.	Affective attitude	8 (5.4)	7 (4.7)	133 (89.9)
My pharmacist is accessible to me.	Burden	9 (6.1)	14 (9.4)	124 (83.8)
I believe I will face stigma or discrimination when receiving services in pharmacies.	Burden	94 (63.5)	39 (26.4)	15 (10.1)
I have consistent access to the same health care professional (e.g., family physician) for my medication-prescribing needs.	Burden	48 (32.4)	21 (14.2)	79 (53.4)
Having PrEP medication available through pharmacist prescribing would benefit my community.	Ethicality	4 (2.7)	3 (2)	141 (95.3)
I believe that pharmacists should be able to prescribe PrEP.	Ethicality	6 (4.1)	7 (4.7)	135 (91.2)
Pharmacists work closely with other health care professionals as part of a team to care for people who take PrEP.	Intervention coherence	10 (6.8)	26 (17.5)	111 (75)
Receiving PrEP from a pharmacist will damage my relationship with my other health care professionals.	Opportunity cost	135 (91.1)	10 (6.8)	3 (2)
Pharmacist prescribing of PrEP will have negative consequences for me.	Opportunity cost	136 (91.9)	7 (4.7)	4 (2.7)
I am worried about the privacy offered by the pharmacy when discussing PrEP with the pharmacist.	Opportunity cost	100 (67.5)	18 (12.2)	30 (20.3)
Pharmacists are knowledgeable about PrEP therapy.	Perceived effectiveness	18 (12.2)	54 (36.5)	73 (49.3)
Pharmacists are able to interpret laboratory tests.	Perceived effectiveness	9 (6.1)	40 (27)	96 (64.9)
I am confident in my ability to visit a pharmacy to receive PrEP.	Self- efficacy	13 (8.8)	14 (9.4)	121 (81.8)
I am capable of telling a pharmacist about my needs for PrEP.	Self-efficacy	5 (3.4)	10 (6.8)	132 (89.8)

PrEP, pre-exposure prophylaxis.

* Data were collapsed to *disagree* (*strongly disagree, disagree*), *neutral* and *agree* (*agree, strongly agree*) (*N* = 148). Participants were allowed to skip questions, if desired.

**Table 3:** Identified themes, interpretation and supporting evidence from interview data for participants’ acceptance of pharmacists’ prescribing of PrEP according to the Theoretical Framework of Acceptability constructs

Construct	Theme	Description	Quotes
Affective attitude	Confidence in pharmacists	Patients expected that PrEP prescribing was a service pharmacists could provide.	“I’ve received some really good advice and some really good help from pharmacists in the past for other issues and other things. So I feel very comfortable with pharmacists prescribing PrEP.” (P8)
	Improving access	Patients positively believed that pharmacists can greatly improve access to PrEP in Nova Scotia.	“I think it could make it a lot more accessible for a lot of people. I think it would be very useful, particularly here in Nova Scotia, where physicians seem a little bit harder to come by, and that’s part of the reason why I’m not on PrEP here because I don’t really have access to a physician.” (P7) “Oh, one of the great parts would be accessibility. With the current physician and nurse practitioner shortages and such, getting to have a primary care practitioner can be difficult . . . .” (P12)
Burden	Highly convenient	Having a pharmacist prescribe does not increase patient burden and may improve strain on the health care system.	“So it’s pretty easy for me to get it now, but I don’t think it would make any difference if I had to go to the pharmacy and get a prescription there. You gotta go somewhere to get it anyway so it doesn’t really make a difference.” (P6) “No, it’s probably less burdensome, [pharmacists] are more available. They’re always there.” (P15)
	Care cohesiveness	Patients were concerned about how the process would work with laboratory requisitions.	“There’s an additional requirement for testing and getting blood work done. So I’m not sure that the pharmacist would be able to provide [that] . . . I’m assuming so.” (P3)
Ethicality	Giving patients options	Improving PrEP access to Nova Scotians aligns with patients’ ethics and personal values.	“It’s sort of becoming almost a core expectation—it’s so common now in the community that people have access to PrEP and are on PrEP, that it just makes sense to offer that to more people. I think it’s sort of unethical not to make it widely available.” (P2)
Intervention coherence	Realistic expectations	Patients in general were familiar with the process and requirements of PrEP prescribing including assessment, lab testing, monitoring and follow-up.	“I’m assuming that the pharmacist would have to do an initial kind of discussion with them to determine eligibility. Obviously, they’re going to have to request a blood test regularly as we have to do anyway, and explain all of that before actually prescribing it. And I’m assuming that there would be like a follow-up appointment with the pharmacist to get the prescription.” (P4)
	Knowledge and education	In addition to prescribing PrEP, patients expect pharmacists to be educated and provide education surrounding sexual health.	“I would say the biggest thing is education. And I think knowing that with PrEP, you have to be tested regularly. You can still get other STIs or STDs and making people aware of that, I think, is a little more work and responsibility for the pharmacist, but I think it’s something that most are willing or would be able to do without any problems.” (P14)
Opportunity cost	No additional costs	Overall, patients did not anticipate losing or avoiding care and relationships with other care providers. They felt confident in their ability to continue to seek care for needs outside of PrEP.	“I don’t necessarily think [it would interfere with other care]. The reason why is because your physician is there for many different reasons and going to see them and with the strain that is on the physicians at this time with the amount of care and other things that they need to be seen for, I don’t think it would necessarily decrease the visits to a physician.” (P10) “So, to take one thing away from my physician, I don’t think I’m losing anything by not being able to interact with him or his staff. I think that it actually is more convenient to be able to talk to my pharmacist who I am extremely comfortable with and very happy to talk to.” (P12)
Perceived effectiveness	Pharmacists are capable	Patients believe that pharmacists are capable of prescribing PrEP and that this service is already within their scope of practice.	“Pharmacists are the experts in the pharmaceutical world. So, you know, I feel like they would understand a lot about it. Both the side effects and the importance of taking it all the time. So, I think they’d be just as effective as MDs, if not more effective.” (P6) “Very effective, just the same way as other programs we have shown to be very effective. I have no areas of concerns. I mean, it’s been proven, you know, we’ve had a few different areas where pharmacists have been very successful at [taking on] more tasks and duties.” (P15)
	Worried about stigma	Patients were concerned about facing stigma from pharmacists, among other health care providers, when receiving sexual health care.	“OK, [pharmacists] might have a general sense of how it works, but are they going to be able to answer specific questions? Again, are they going to treat the populations who are looking to get this medication equitably? I don’t know. So, I definitely have some concerns there, but in fairness, it’s my same concern for any medication that any person who is marginalized might be attempting to access.” (P5) “I guess the only hesitation I have would just be, pharmacists like any health care professional would be subject to their own biases and preferences that are highly associated with the trans community. I guess there would be concern that a pharmacist would either refuse to prescribe or not prescribe in a supportive and nonjudgmental way, but again that’s sort of ubiquitous in health care and I don’t know how adding pharmacists would be a negative.” (P9) “The only thing I worry about is stigma, what kind of questions am I going to be asked in public in order to obtain this medication.” (P5)
Self-efficacy	Comfortable setting	Patients are excited about increasing access to PrEP in Nova Scotia and feel very strongly about increasing avenues for patients to obtain it. Having pharmacists prescribe does not increase barriers to patients accessing PrEP.	“PrEP is a drug that is a privilege to have and improving its access is important.” (P1) “Yeah, none. There would be fewer barriers as a result, this program would lower the challenges to accessing this critical medicine.” (P2)
	Team coordination	Patients are willing to work with their pharmacist and other health care providers to obtain PrEP therapy.	“100%. I could see myself getting it from a pharmacist. The only barrier I could see would be the bloodwork aspect of it. If that was a possible barrier, other than that I feel like it would be very easy, very simple and I don’t see any issues at all.” (P6)

PrEP, pre-exposure prophylaxis; STD, sexually transmitted disease; STI, sexually transmitted infection.

### Construct 1: Affective attitude

The results from the Affective Attitude construct of the survey indicated that 92.6% of respondents would feel comfortable having a pharmacist prescribe them PrEP and that another 89.9% of respondents were comfortable talking with pharmacists about PrEP before starting therapy. Two themes were identified from the qualitative analysis for this construct and represented overwhelmingly positive attitudes toward PrEP prescribing: “Confidence in pharmacists” and “Improving access.” Themes encompassed participants’ trust in pharmacists and their abilities as well as the need to increase access to PrEP in Nova Scotia.

### Construct 2: Burden

Most respondents (83.8%) felt that they had access to pharmacists, whereas only 53.4% of respondents felt that they had the same access to a health care professional for prescribing their routine medications. While many (63.5%) respondents did not feel that they would face stigma in pharmacies, approximately one-third of respondents felt neutral or agreed that they would. The qualitative analysis identified 2 major themes for burden: “Highly convenient” and “Care cohesiveness.” Participants felt pharmacy-based services would streamline the process and even reduce strain on the health care system. Some participants were concerned about delivery of care being cohesive, specifically addressing concerns if pharmacists are unable to order laboratory tests.

### Construct 3: Ethicality

Respondents felt strongly that having PrEP available through pharmacies would benefit the community (95.3%). In addition, 91.2% of respondents felt that pharmacists should be able to prescribe PrEP. One major theme was identified from the qualitative data, “Giving patients options.” This theme encompassed the value that participants wanted convenient options to access PrEP to reduce risks of HIV transmission throughout the community.

### Construct 4: Intervention coherence

Most (75%) respondents were aware that pharmacists work closely with other health care professionals to provide care for patients taking PrEP. From the qualitative analysis, 2 themes were identified: “Realistic expectations” and “Knowledge and education.” Interviewees were generally familiar with the process of PrEP prescribing, including assessment, requirement for laboratory testing, routine follow-up and monitoring with the prescriber. However, they also felt that pharmacists should be providing more education surrounding sexual health and laboratory testing if prescribing PrEP.

### Construct 5: Opportunity cost

More than 90% of respondents (91.1%) disagreed that having a pharmacist prescribe PrEP would damage their relationship with other health care providers. Similarly, 91.9% of respondents disagreed that there would be negative consequences of pharmacist prescribing of PrEP. A total of 32.5% were neutral or agreed that they would be worried about privacy offered in a pharmacy. The qualitative analysis identified 1 theme, “No additional costs.” Participants did not express worry about accessing PrEP through a pharmacist and viewed it as an option that may decrease strain on the health care system.

### Construct 6: Perceived effectiveness

Approximately half (49.3%) of respondents agreed that pharmacists were knowledgeable about PrEP. More respondents (64.9%) agreed that pharmacists had the ability to interpret laboratory tests. The qualitative analysis identified 2 themes, “Pharmacists are capable” and “Worried about stigma.” Although participants were highly supportive of pharmacists prescribing PrEP and believed them to be capable, concerns were raised about the possibility of experiencing stigma from pharmacists when accessing PrEP services. Participants believed that experiencing stigma in pharmacies would decrease the effectiveness of a PrEP-prescribing service.

### Construct 7: Self-efficacy

Most (81.8%) respondents were confident in their own ability to visit a pharmacy to access PrEP therapy, with 89.8% of respondents feeling comfortable telling a pharmacist about their PrEP needs. Two themes were identified from the qualitative analysis, “Comfortable setting” and “Team coordination” (Table 3). Participants were confident and comfortable obtaining PrEP from a pharmacist based on previous positive experiences with pharmacists. In addition, participants believed they would be able to coordinate their overall health care needs between health care providers, including pharmacists.

## Discussion

This study found that target PrEP users were highly accepting of a PrEP-prescribing service by pharmacists in Nova Scotia. They have the confidence in pharmacists to deliver this service and believe it will increase PrEP access and benefit their communities. Similar results were obtained from the qualitative interviews, with most results supporting the role of the pharmacist as a PrEP prescriber.

Although respondents were confident with pharmacists’ abilities to prescribe PrEP, they did identify privacy and stigma concerns when accessing PrEP-prescribing services. These findings align with other studies that highlighted these concerns for pharmacy-based PrEP or sexually transmitted infection–testing services.^10,18^ While privacy may be addressed using consultation rooms, pharmacists should be conscious of the sensitive nature of PrEP, especially when addressing patients in public areas. In addition, respondents’ concerns about stigma, whether actual or perceived, may result in stress for these patients and could result in care delay or avoidance.^10,11,23^ The profession should therefore work collectively to ensure pharmacies are inclusive and affirmative in the services they provide.

A second key finding was that respondents believed that a PrEP-prescribing service by pharmacists would not interfere with their relationships with other health care professionals. This is especially important for those eligible for PrEP in Nova Scotia, as they typically belong to underserved populations without consistent access to care. Issues with access to care were further demonstrated in the study results in that just over half of respondents (53.4%) had access to nonpharmacist prescribers, but 83.8% had access to a pharmacist. Given the ongoing primary care provider shortage in Nova Scotia, with 11.4% of Nova Scotians on the “Need a Family Practice Registry” as of October 2022, pharmacists can provide much needed access to PrEP for those without providers as well as provide PrEP to those with other providers without interfering with those patient-provider relationships.^24^ In keeping with the prescribing standards, collaboration with those in the patient’s circle of care would remain a consistent feature of PrEP prescribing, similar to any other prescribing activity.

This study has implications for both practice and research. Based on these findings, PrEP prescribing should be considered as part of pharmacists’ prescribing scope of practice in Nova Scotia. The acceptance and demand from target users exist, and it appears to be perceived as an effective way to increase PrEP access in convenient and safe environments. More work needs to be done to ensure that pharmacists have the appropriate scope (e.g., laboratory test ordering and viewing) to provide comprehensive care. Engaging in business case planning around PrEP services as part of preventative health service delivery in pharmacies will also facilitate implementation. With respect to future research, this study identified that acceptance may be dependent on the ability of pharmacists to offer private and stigma-free environments for PrEP-related services. Future work should be conducted to determine the most effective ways to reduce these concerns and ensure that all patients can be confident in their ability to receive safe and confidential care.

This study has limitations that should be addressed. First, the study was limited to 1 Canadian province, and most respondents were located within the capital city area; thus, the results may not reflect the attitudes and experiences of those in more rural areas. Pharmacists in Nova Scotia have some of the most extensive prescribing authority within the country, and this may influence the generalizability of the findings to other provinces and regions. Second, although PrEP is indicated for 3 populations in Nova Scotia, most of the respondents identified as MSM or TGW. While this likely reflects the majority of the population for which PrEP is indicated in Nova Scotia, findings may not be attributable to people who inject drugs and/or heterosexual persons with HIV-positive partners. It is also possible that the sampling strategy via social media and e-mail was less accessible to people who inject drugs, and this should be considered for future studies. Finally, most respondents were either current users or very interested in PrEP, which may have skewed results in favour of increasing PrEP access to the community.

## Conclusion

This study found that pharmacist prescribing of PrEP is acceptable to target users in Nova Scotia. Respondents were accepting across all 7 assessed constructs yet identified concerns related to privacy in pharmacies and stigma that may be encountered by pharmacists. The feasibility of PrEP prescribing by pharmacists should be pursued as an intervention to increase access to this important medication. ■
